# Simultaneous Localization and Map Change Update for the High Definition Map-Based Autonomous Driving Car

**DOI:** 10.3390/s18093145

**Published:** 2018-09-18

**Authors:** Kichun Jo, Chansoo Kim, Myoungho Sunwoo

**Affiliations:** 1 Department of Smart Vehicle Engineering, Konkuk University, Seoul 05029, Korea; kichun.jo@gmail.com; 2 Department of Automotive Engineering, Hanyang University, Seoul 04763, Korea; chansoo7857@gmail.com

**Keywords:** high definition (HD) map, autonomous cars, map change detection, cloud map, localization

## Abstract

High Definition (HD) maps are becoming key elements of the autonomous driving because they can provide information about the surrounding environment of the autonomous car without being affected by the real-time perception limit. To provide the most recent environmental information to the autonomous driving system, the HD map must maintain up-to-date data by updating changes in the real world. This paper presents a simultaneous localization and map change update (SLAMCU) algorithm to detect and update the HD map changes. A Dempster–Shafer evidence theory is applied to infer the HD map changes based on the evaluation of the HD map feature existence. A Rao–Blackwellized particle filter (RBPF) approach is used to concurrently estimate the vehicle position and update the new map state. The detected and updated map changes by the SLAMCU are reported to the HD map database in order to reflect the changes to the HD map and share the changing information with the other autonomous cars. The SLAMCU was evaluated through experiments using the HD map of traffic signs in the real traffic conditions.

## 1. Introduction

The autonomous cars are one of the important future technologies that will change the paradigm of the automotive and transportation industry. The realization of the autonomous car can allow the human driver to reduce the burden of driving and prevents the accident caused by the driver carelessness. In addition, the autonomous cars managed by the intelligent transportation system can improve the traffic flow and optimize the energy consumption. For the autonomous driving of the cars, the autonomous driving system must first understand the surrounding environment. Then, it can determine the optimal behavior and trajectory and control the vehicle to follow the planned behavior and trajectory [1].

Perception sensors such as camera, radar, and lidar can provide the nearby environmental information of the autonomous car based on the sensor data processing. However, the current perception technologies of the data processing have constraints to detect the all surrounding environment because of the limitations of sensor visibility range and recognition performance. To overcome the limitations, the pre-built environmental map can be used to obtain the environmental information, which is called High Definition (HD) map. The HD map contains the several physical information on the roads, such as lanes, traffic signs, traffic lights, barriers, and road surface marking, within the 10–20 cm accuracy [2]. By searching the nearby physical features on the HD map, the autonomous car can access the information without the perception processing and the sensor visibility limitations.

However, there is always a possibility that the physical environments on the HD map are changed because new physical features are added, or the physical features saved on the HD map disappear or move. The changes of the physical features which are not reflected on the HD map can cause the unexpected problems for the autonomous driving due to the incorrect understanding of the surrounding environmental information. For example, the planning system can make an incorrect decision about the behavior and trajectory due to the use of HD map that has not been updated. The localization of the autonomous car might estimate the inaccurate position due to the landmark misalignment between the perception and the HD map. To prevent these problems, the changes of HD map must be detected and managed to keep up-to-date road information.

This paper proposes a simultaneous localization and map change update (SLAMCU) algorithm to detect the HD map changes and to update the changes to the HD map database. The SLAMCU does not use the special mapping equipment for the map updating, but it uses the onboard sensors of autonomous cars (or intelligent vehicles), such as perception sensors (camera, lidar, and radars) and vehicle motion sensors (a wheel speed sensor, a steering angle sensor, and an inertial measurement unit). For the detection of the map changes, the SLAMCU algorithm uses the evidence (Dempster–Shafer) theory for the reasoning of the HD map existence. The HD map existence can be updated by Dempster combination rule based on the detection confidence and the field-of-view (FoV) configuration of the perception sensors. The map changes can be classified into three classes including the normal, delete, and new based on the results of existence inference. For the normal HD map, the SLAMCU algorithm performs a localization that estimates the vehicle position. The delete HD maps are excluded for the localization update. For the new map feature, the SLAMCU execute a SLAM (Simultaneous Localization and Mapping) that estimate the position of the map and vehicle simultaneously. A Rao–Blackwellized particle filter (RBPF) is used to concurrently perform the localization and the SLAM. The detected and updated map changes by the SLAMCU of the individual car can be uploaded to the map database of the HD map provider in order to reflect the map changes to the HD map and share with the other autonomous and intelligent vehicles.

This paper is organized as follows. Section 2 presents the definition of the HD and the problems of its changes. Section 3 introduce the SLAMCU algorithm, and Section 4 explains the map change management system. Section 5 describes the implementation of SLAMCU based on the RBPF, and Section 6 provides verification of the SLAMCU based on the experiences. The final section provides conclusion and future works.

## 2. High Definition (HD) Map for Autonomous Cars

### 2.1. High Definition (HD) Map

The autonomous cars require the surrounding environment information, such as objects, traffic control devices, and roadway geometry, in order to perform the planning and control. Perception sensors, such as cameras, radars, and lidars, can provide the information of surrounding environments in real time, but the sensors have limitations for the perception range and recognition performance. The perception sensors cannot detect the physical environments that are located far from the ego-vehicle or are blocked by obstacles. Also, not all physical environments on the road can be recognized using the current perception technology. To overcome the limitation of perception, we can apply a HD map for the autonomous cars [3,4,5].

The High definition (HD) map is a detailed representation of the physical environment features, which the autonomous cars can use for autonomous driving. The HD map can be called a Highly autonomous driving (HAD) map or a precise map, but we unified the terminologies into the HD map in this paper. The compositions of the HD map classified into three types of map feature: static object, traffic control devices, and roadway geometry. The static object represents something that can be collided with the ego-vehicle, such as buildings, walls, trees, poles, and barriers. The traffic control devices can provide information about traffic rules that must be followed on the road, such as road surface markings, speed bump, traffic signs, and traffic lights. The roadway geometry provides information that should be followed by vehicles to reach the desired destination and can be represented by polylines, polynomial curves, or splines.

The autonomous car can utilize the HD map for autonomous driving by accessing information about the surrounding environment stored on the map. To access the surrounding environment information on the map, the current pose (position and heading) of the ego-vehicle must be provided to the searching engine of the HD map. Therefore, a localization algorithm that estimates the current vehicle pose is an essential component for the intelligent driving system using the HD map. The localization is able to estimate the pose using a dead reckoning (DR) of vehicle motion sensors or global navigation satellite system (GNSS). The HD map can also be used for accurate localization by aligning landmark perception with the HD map landmarks [6,7,8]. For instance, the autonomous car can estimates the position of ego-vehicle by matching the traffic sign perception from the camera and the traffic sign position data in the HD map. The landmarks must be static features that are recognized by sensors and saved in the HD map, such as buildings, walls, trees, poles, traffic sign, traffic light, and lanes.

### 2.2. Previous Studies

**Figure 1:**
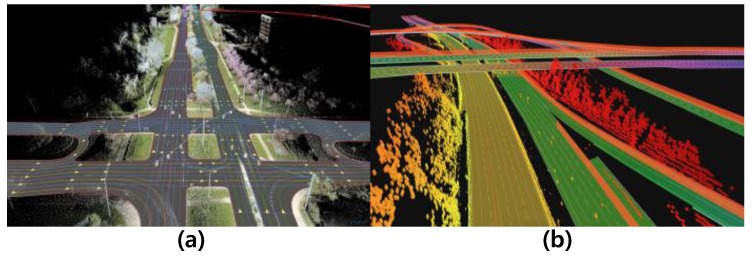
Examples of the HD map. (**a**) HERE (**b**) TomTom.

There were many previous studies to use the HD map for the autonomous driving. A route network definition file (RNDF) was used as the HD map in the DARPA challenges to provide the routes to autonomous cars [9]. Google self-driving cars used the 3D high-accurate HD map [10]. The demonstration in Bertha Benz Memorial Route by the Daimler utilized a Lanelets that is an efficient data structure for the drivable environment map as the HD map [11]. Not only these studies, there have been many autonomous car tests based on the HD map [12,13,14,15,16,17,18,19]. These previous studies are evidence that the HD map is a critical factor in the future industry of autonomous. A technical report [20] also forecasts that the HD map is one of the key technical components of the autonomous car era. Therefore, many mapping companies, such as HERE [21] and TomTom [22], are preparing or starting to provide the HD map for the era of autonomous cars. HERE provides a HD map that contains various semantic features, such as road geometry, lane boundaries, barriers, traffic signs, and traffic lights, in 10-to-20 cm accuracy, as shown in Figure 1a. TomTom also provides semantic features similar to HERE and provides depth information for static objects via Road DNA [23], as shown in Figure 1b.

A ground mapping is mainly used for the HD map building due to the high accuracy and reliability although aerial mapping is much cheaper and faster. The process of ground mapping consists of three steps: a data acquisition, a data processing, and a database management. The data acquisition is a process to collect the information about the physical environments by surveying with special mapping vehicles equipped with various mapping sensors such as a real-time kinematic (RTK)-GNSS, an inertial measurement unit (IMU), cameras, and lidars. After the data acquisition, the HD map features (static object, traffic control devices, and roadway geometry) are extracted through the data processing. The final step is the database management for providing a service of the map management and access. Due to these series of multiple processes, the mapping cost of HD map is much higher than the mapping of the topological map that is used for the in-car navigation module.

### 2.3. Problems of HD Map Changes

The most significant problem with the HD map is the change in the physical features. The change of physical feature can give the incorrect environment information to the autonomous driving system, and it can cause a negative impact on the safe autonomous driving. The localization based on the alignment of landmark perception and HD map also can be suffered from degradation of the accuracy and reliability due to a misalignment by the map changes. Therefore, the changes of HD map must be detected and managed by the autonomous driving system to prevent the performance degradation.

**Figure 2:**
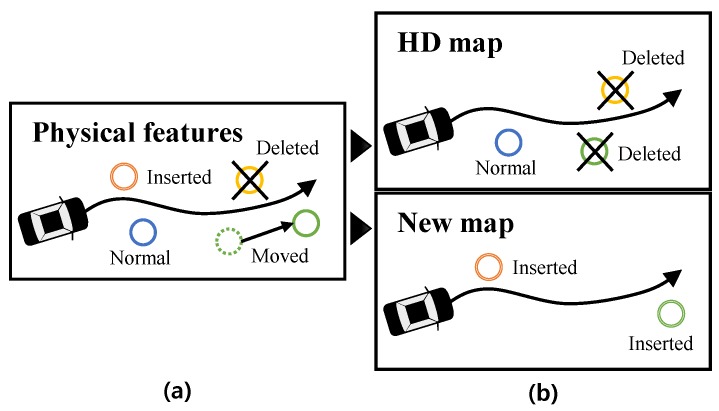
Definition of the HD map changes. (**a**) physical world. (**b**) HD map $m_{HD}$ and new map $m_{new}$. The map component can be classified into $m_{HD\{normal\}}$, $m_{HD\{delete\}}$, $m_{HD\{insert\}}$.

To detect and manage HD map change issues, we must clearly define what the HD map change is. There are three cases for the changes of the physical features on the road as shown in Figure 2a: (1) a physical feature is moved; (2) a new physical feature is inserted; and (3) the physical feature is deleted. Since (1) the movement of physical features can be described as a series of (3) the deletion of physical feature and (2) the insertion of the new physical feature, we can define the changing physical feature as two classes that are a new physical feature and deleted physical feature. To reflect the physical feature changes to the HD map-based autonomous driving, we can classify the map features into three classes. The map features on the HD map can be classified into (1) normal map feature $m_{HD\{normal\}}$ and (2) deleted map feature $m_{HD\{delete\}}$. To deal with the insertion of the new physical feature, a new map is required to manage the (3) new map feature $m_{new}$, as shown in Figure 2b.

## 3. Simultaneous Localization and Map Change Update(SLAMCU) for HD Map

### 3.1. Management of the HD Map Changes by Individual Autonomous Vehicles

There are two ways to keep the up-to-date HD map that reflects physical feature changes. The first method is applying the ground mapping based on the special mapping vehicles to the map change detection and update. The special mapping vehicles survey all roads to collect the mapping data, the map feature changes are detected in the data processing step, and the changed map features are uploaded to map database. However, the ground mapping-based map change update is too expensive and not agile because it is based on the special mapping vehicles with limited frequency and range of operation.

The second way to recognize and update the HD map changes is to use the perception and localization of individual autonomous cars (or intelligent vehicles with the perception and localization capabilities) driving on the road. This method is more cost-effective than the first because there is no need to operate special mapping vehicles to monitor map changes. In addition, the HD map can keep up to date with the latest information because the map change update can be performed by multiple autonomous vehicles at the same time.

**Figure 3:**
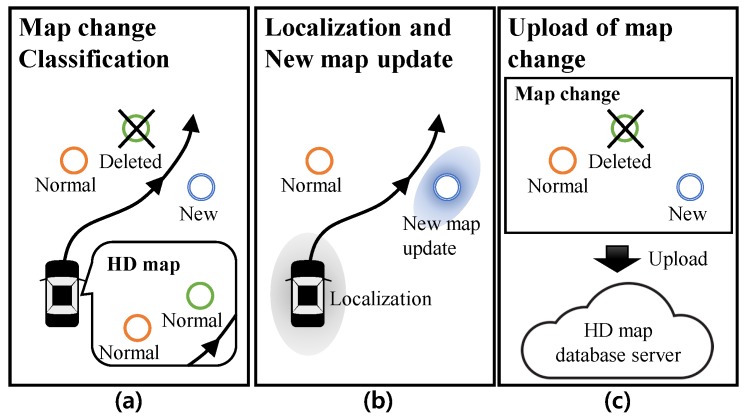
Process of the map change update. The first step is map change classification, the second step is the utilization the HD map and the updating the new map features, and final step is the upload to HD map database server.

The process of the map change update based on the individual autonomous cars can be divided into three steps as shown in Figure 3. The first step is a map change classification based on the physical features perception and the localization. The map features in the HD map is classified into the normal HD map feature $m_{HD\{normal\}}$ and the deleted HD map feature $m_{HD\{delete\}}$. The new physical features that are not in the HD map are classified to the new map feature $m_{new}$. The second step is to utilize the classified features into the autonomous driving. The $m_{HD\{normal\}}$ is used for the autonomous driving and localization of autonomous vehicles. Conversely, the $m_{HD\{delete\}}$ has no effect on the autonomous driving and localization. For the new map feature $m_{new}$, the autonomous car should estimate the state (position) of the $m_{new}$, concurrently use for the autonomous driving and localization. The algorithm for the first and second step is Simultaneous Localization and Map Change Update (SLAMCU). The final step is reporting and uploading the map changes to the HD map database server (map provider). For the updating to the map server, we can apply a standard map update protocol such as SENSORIS [24].

However, there are constraints of the individual vehicles based map change update due to the performance limitation of localization and perception on the vehicles. The perception limitation can cause the misclassification of the map feature changes, and the state estimation of new map features have poor accuracy compared to the mapping with special mapping vehicles. Nevertheless, the map change update based on the individual vehicles is worth because it can provide the probable location of map changes to the map provider, and it can supply the temporary information of the new map features that can be used before the mapping with the special mapping vehicles.

### 3.2. Analysis the SLAMCU Using Graph Structure

The simultaneous localization and map change update (SLAMCU) has two major functional requirements. The first requirement is to classify the HD map features into the normal $m_{HD\{normal\}}$ and the deleted $m_{HD\{delete\}}$, and to only apply the normal feature $m_{HD\{normal\}}$ for the localization and autonomous driving. The second functional requirement is to find the unregistered new physical feature, to register the found physical feature to new map feature $m_{new}$, to estimate the state of the $m_{new}$, and to apply the $m_{new}$ to the localization and autonomous driving. To design and implement the functional requirements of the SLAMCU based on the probabilistic framework, we analyze the SLAMCU problem using the graph of dynamic Bayesian networks (DBN).

A directed graph of a DBN based on the Markov process assumption can represent the various types of localization and mapping problems using the probabilistic framework. The DBN is composed of nodes for probabilistic random variables and directed edges for representing conditional dependencies between two nodes. The nodes of the localization and mapping problems consist of the state, map, input, and measurement. The $state\;x_{1:t}=\left\{x_{1},\cdots ,x_{t}\right\}$ indicates a sequence of the vehicle pose from discrete time steps 1 to *t*. The $input\;u_{1:t}=\left\{u_{1},\cdots ,u_{t}\right\}$ represents the vehicle motion input, and the $measurement\;z_{1:t}=\left\{z_{1},\cdots ,z_{t}\right\}$ represents the observation of the map features by perception sensors. The mapm indicates the position of map features that can be observed by the perception of autonomous cars. The edges that represent probabilistic constraints between the nodes consists of a statetransitionmodel and measurementmodel for the localization and mapping problems. The $state\;transition\;model\;p(x_{t}|x_{t-1},u_{t})$ represents a motion constraint between the two adjacent vehicle state $x_{t}$ and $x_{t-1}$ regarding the motion control input $u_{t}$. The state transition model can predict the future vehicle state $x_{t}$ using the previous state $x_{t-1}$ and input $u_{t}$. The $measurement\;model\;p(z_{t}|x_{t},m_{t})$ describes the probability of measurement $z_{t}$ given the state $x_{t}$ with the map *m*. The measurement model can estimate the probability distribution of the measurement $z_{t}$ based on the current state $x_{t}$ and map features *m*.

**Figure 4:**
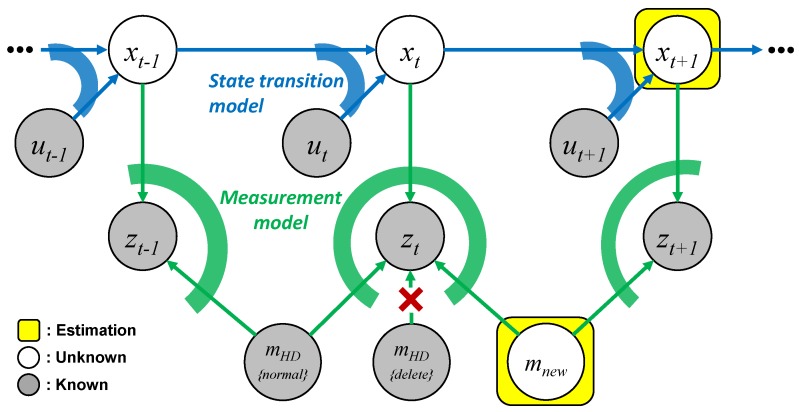
dynamic Bayesian networks (DBN) Graph representation of the SLAMCU.

The SLAMCU problem can be interpreted by graph structure as shown in Figure 4. The known nodes are input $u_{1:t}$, measurement $z_{1:t}$, and the map features $m_{HD\{normal,delete\}}$ on HD map. The unknown nodes are state $x_{1:t}$ and new map feature $m_{new}$. The SLAMCU wants to estimate the recent state $x_{t+1}$ and the new map feature $m_{new}$ based on the known nodes and the edge constraints. The first functional requirement of the SLAMCU can be represented through the graph structure as a localization problem with a data association. The localization estimates the state $x_{1:t}$ using the input $u_{t}$, measurement $z_{t}$, and the HD map features $m_{HD\{normal,delete\}}$. The existence inference-based map management system generates links between the measurement $z_{t}$ and the normal HD map features $m_{HD\{normal\}}$ by the edge of the measurement model, and disconnects the $z_{t}$ to the deleted HD map features $m_{HD\{delete\}}$. The second functional requirement can be interpreted as a SLAM (Simultaneous Localization and Mapping) problem that concurrently estimate the state $x_{t}$ and the new map feature $m_{new}$. The existence inference-based map management generates a new link between the measurement $z_{t}$ and the new map features $m_{new}$ by the measurement model edge. In conclusion, the SLAMCU can be designed and implemented by the combination of localization and SLAM with the map management based on the existence inference of the physical features.

## 4. Map Management of SLAMCU

### 4.1. Functional Objectives of the Map Management

**Figure 5:**
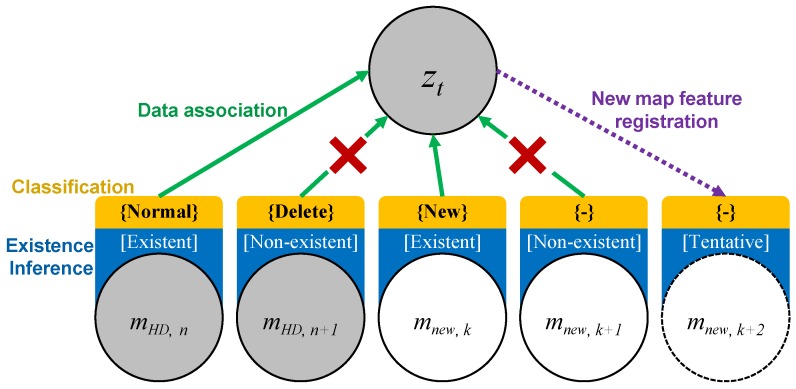
Process of the map change update.

A map management method classifies the map changes and performs the data association between measurement and map. The overall structure of the map management system is described in Figure 5. First, the map management finds the new physical features that are not in the HD map $m_{HD}$ based on the measurement $z_{t}$ with inverse measurement model and registers the new feature into the new map $m_{new}$. Second, the existence of the each map feature in the HD map $m_{HD}$ and the new map $m_{new}$ is inferred based on the evidential approach (Dempster–Shafer theory). The existence represents whether the physical feature really exists in the position of the corresponding map feature. The existence of each map feature is represented by three states of [existent, non-existent, tentative]. Third, the map features in the HD map $m_{HD}$ and new map $m_{new}$ are classified into the three classification types of normal, deleted, and new based on the result of existence inference. The map features classified into the deleted and new are reported to the map database as the changed map features. Finally, the data association is performed based on the result of map classification types. The only normal HD map feature and new map features are associated with the measurement $z_{t}$ for the SLAMCU update.

### 4.2. Existence Inference Based on the Evidence Theory

#### 4.2.1. Dempster-Shaper Theory for the Existence Inference

The existence of the map feature is an important factor to classify the map type and determine the data association. The map existence represents the possibility that real physical features exist in the map feature location stored on the map. Probabilistic approaches can be applied to infer the map existence. If the physical feature is *non-existent* in the map feature position, the map existence probability of a map feature is zero. On the other hand, the map existence probability is one if the real physical feature is existent in the map feature position. However, the probabilistic approach cannot explicitly handle the existence of tentative (unknown), indicating an unclear situation due to the insufficient measurement updates. Also, the conflict existence, which represents a situation about different measurements for the same map feature, is ambiguous to describe using the probability approach. The probability of the both tentative and conflict are denoted by 0.5 using the probabilistic approach, but it is unsuitable to explicitly represent the both situations.

To deal with the tentative (unknown) and conflict state, Dempster–Shafer (DS) theory can be applied to infer the existence of the map features on the HD map $m_{HD}$ and new map $m_{new}$. The two states of Exist∃ and Non−exist∄ for the map feature existence are determined as a frame of discernment Ω={∃,∄}. The existence based on the DS theory takes into account the power set $2^{\Omega }=\left\{\phi ,\exists ,∄,\Omega \right\}$, which is the set of all subsets of the Ω={∃,∄}. This means that the DS-based existence inference can consider tentative state of Ω (both ∃ and ∄ are possible) and conflict state ϕ (the ∃ and ∄ are conflict each other) explicitly.

For each existence state in the power set, a mass function mass can be applied to quantify the evidence of the existence. The mass functions of mass(∃) and mass(∄) represent the evidence of the existence states is existent and non−existent, respectively. The mass function of mass(Ω), which considers the union of ∃ and ∄, represents the existence is tentative (unknown), and mass(ϕ) represents the existence state is conflicted by a different inference information. The sum of mass functions for the power set should be one based on the definition of the mass function in the DS theory.

The mass functions of the measurement $z_{t}$ for the elements of the power set at time *t* can be determined based on the existence confidence λ of the measurement. The existence confidence λ represents a belief level between [0, 1] of measurement $z_{t}$ for the perception of physical landmark features that are located in the perception field-of-view (FoV). If the map features are located in the perception FoV and are detected to the measurement $z_{t}$, then the mass functions of the measurement at time *t* can be represented by

(1) $$\begin{array}{cc} mass_{z_{t}}\left(\phi \right)=0,\; & mass_{z_{t}}\left(\exists \right)=\lambda ,\\ mass_{z_{t}}\left(∄\right)=0,\; & mass_{z_{t}}\left(\Omega \right)=1-\lambda \end{array}$$

On the other hand, if the map features are located in the FoV but the physical feature for the corresponding map feature is not detected by the measurement $z_{t}$, then the mass functions of the non-measurement can be represented by

(2) $$\begin{array}{cc} mass_{z_{t}}\left(\phi \right)=0,\; & mass_{z_{t}}\left(\exists \right)=0,\\ mass_{z_{t}}\left(∄\right)=\lambda ,\; & mass_{z_{t}}\left(\Omega \right)=1-\lambda \end{array}$$

#### 4.2.2. Inference of the Map Feature Existence Based on the Dempster Combination Rule

The instant mass functions of the measurement $mass_{z_{t}}$ at time *t* cannot be directly used to determine the map features existence of the HD map $mass_{HD_{t}}$ and new map $mass_{new_{t}}$. This is because the measurement can contain uncertainty due to the sensor noise and limitations of recognition. Therefore, the measurement existence should be incrementally integrated to the existence of the corresponding map feature $mass_{HD_{t}}$ and $mass_{new_{t}}$. The initial mass functions of HD map features with index *i* can be defined based on the existence confidence of HD map feature $\lambda_{HD}$, as described in

(3) $$\begin{array}{cc} mass_{HD_{0}\left\{i\right\}}\left(\phi \right)=0,\; & mass_{HD_{0}\left\{i\right\}}\left(\exists \right)=\lambda_{HD},\\ mass_{HD_{0}\left\{i\right\}}\left(∄\right)=0,\; & mass_{HD_{0}\left\{i\right\}}\left(\Omega \right)=1-\lambda_{HD} \end{array}$$

The mass functions of new map features with index *j* can be initialized by the vacuous mass function:

(4) $$\begin{array}{cc} mass_{new_{0}\left\{j\right\}}\left(\phi \right)=0,\; & mass_{new_{0}\left\{j\right\}}\left(\exists \right)=0,\\ mass_{new_{0}\left\{j\right\}}\left(∄\right)=0,\; & mass_{new_{0}\left\{j\right\}}\left(\Omega \right)=1 \end{array}$$

that represents the no prior information. After the initialization at t=0, the measurement existence $mass_{z_{t}}$ is incrementally accumulated into the map feature existence $mass_{HD_{t}}$ and $mass_{new_{t}}$. Dempster combination rule ⊕ of evidence theory is used to accumulate the measurement existence $mass_{z_{t}}$ to the each map feature existence at time t−1, as described in

(5) $$\begin{array}{cc} mass_{HD_{t}\left\{i\right\}} & =mass_{HD_{t-1}\left\{i\right\}}⊕mass_{z_{t}}\\ mass_{new_{t}\left\{j\right\}} & =mass_{new_{t-1}\left\{j\right\}}⊕mass_{z_{t}} \end{array}$$

The Dempster combination rule ⊕ can be constructed based on the Dempster normalization

(6) $$\begin{array}{cc} mass_{1⊕2}\left(A\right) & =\frac{mass_{1\cap 2}\left(A\right)}{1-mass_{1\cap 2}\left(\phi \right)},\forall A\subseteq \Omega ,A\neq \phi \\ mass_{1⊕2}\left(\phi \right) & =0 \end{array}$$

with the conjunctive combination rule

(7) $$\forall A\subseteq \Omega ,mass_{1\cap 2}\left(A\right)=\sum_{B\cap C=A|B,C\subseteq \Omega }mass_{1}\left(B\right)\;\cdot \;mass_{2}\left(C\right)$$

### 4.3. Process of the Map Management

The process for the implementation of the functional objectives (Figure 5) consists of four steps: a pre-data association, an updating the map feature existence, a classification of map changes, and a data association.

#### 4.3.1. Pre-Data Association

**Figure 6:**
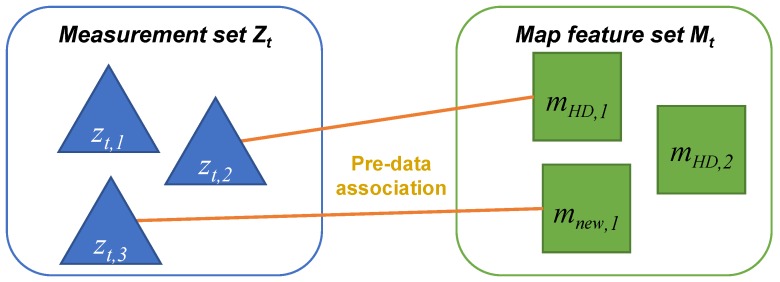
Pre-data association between the measurement set and map feature set.

The first step is pre-data association between the measurement set $Z_{t}=\left\{z_{t,1},z_{t,2},\cdots \right\}$ and the map feature set $M_{t}=\left\{m_{HD,1},m_{HD,2},\cdots ,m_{new,1},m_{new,2},\cdots \right\}$ located in the sensor FoV, as shown in Figure 6. In the pre-data association, each measurement is associated with each map feature by maximizing the likelihood of the measurement $z_{t}$ with a certain likelihood threshold.

#### 4.3.2. Updating the Map Feature Existence

There are three types of pre-data association: (1) unassociated measurement [$z_{t},1$]; (2) associated measurement-map feature [$z_{t,2}-m_{HD,1}$, $z_{t,3}-m_{new,1}$]; and (3) unassociated map feature [$m_{HD,2}$]. The unassociated measurements are registered as the new features into the new map $m_{new}$ with the existence initialization using the (4). The map feature existence for the associated measurement-map feature is updated based on the $mass_{z_{t}}$ of (1) using the Dempster combination rule of (5). The map feature existence for the unassociated map feature is updated based on the $mass_{z_{t}}$ of (2).

#### 4.3.3. Classification of Map Changes

The representative existence of each map feature can be determined to be the existence state with the maximum mass function. For the HD map features, the map feature of Existent is classified into Normal class and the Non−existent feature is classified into Delete class. For the new map features, the only Existent map feature is classified into New class, and the others are not classified. The map features classified into the Delete or New class are considered as map changes.

#### 4.3.4. Data Association

The classification result of the map changes is used to the final data association for the updating of the SLAMCU. As shown in Figure 5, the only Normal class of HD map feature and New class of new map feature are associated with the measurement $z_{t}$.

## 5. SLAMCU Based on Rao–Blackwellized Particle Filter

The SLAMCU is a problem to estimate the unknown vehicle pose state $x_{t}$ and new map features $m_{new}$ based on the known control input $u_{1:t}$, measurement $z_{1:t}$, and the HD map features $m_{HD}$. Therefore, the SLAMCU can be represented in the posterior of conditional probability as described in (8).

(8) $$p(x_{1:t},m_{new}|u_{1:t},z_{1:t},m_{HD})$$

The Equation (8) can be factorized into (9).

(9) $$p\left(x_{1:t}|u_{1:t},z_{1:t},m_{HD}\right)p\left(m_{new}|x_{1:t},u_{1:t},z_{1:t}\right)$$

If the $m_{new}$ contains the *N* number of features, the (9) can be represented into (10).

(10) $$p\left(x_{1:t}|u_{1:t},z_{1:t},m_{HD}\right)\sum_{i=1}^{N}p\left(m_{new,i}|x_{1:t},u_{1:t},z_{1:t}\right)$$

A Rao–Blackwellized particle filter (RBPF) can be applied to implement the posterior (10). The RBPF is a combination of the standard particle filter and the Kalman filter. Because of the hybrid characteristics of the RBPF, it is available to take the both advantages of the particle filter and Kalman filter. Therefore, there were many studies to use the RBPF for localization and mapping [25,26,27]. Among these previous studies, FastSLAM is the most successful SLAM implementation based on the RBPF [28]. Therefore, the SLAMCU implementation follows the FastSLAM framework; however, the different things with the FastSLAM are (1) the SLAMCU take into account the HD map and (2) the map management system is needed to manage the HD map changes and new map features.

The SLAMCU based on the RBPF uses particle filter to estimate the posterior of vehicle pose state $p(x_{1:t}|u_{1:t},z_{1:t},m_{HD})$. For the new map feature $m_{new}$, the SLAMCU uses Extended Kalman filter (EKF) to estimate the posterior of $p(m_{new}|x_{1:t},u_{1:t},z_{1:t})$.

The particles $Y_{t}$ in SLAMCU are described as

(11) $$Y_{t}^{\left[k\right]}=<x_{t}^{\left[k\right]},\mu_{1,t}^{\left[k\right]},\Sigma_{1,t}^{\left[k\right]},\cdots ,\mu_{N,t}^{\left[k\right]},\Sigma_{N,t}^{\left[k\right]}>$$

where [k] is the index of particle, $\mu_{n,t}$ and $\Sigma_{n,t}$ are the mean and variance of the Gaussian model of the *n*-th new map feature location. The total number of particles is *M*, so the range of the index *k* is from 1 to *M*. The process of the SLAMCU based on the RBPF consists of four steps: a prediction, an update of a new map feature, an importance weighting, and a resampling.

### 5.1. Prediction

At the prediction step, the new sample state $x_{t}$ of each particle *k* is predicted by applying the state transition model to the previous state $x_{t}$ with control input $u_{t}$, as described in (12).

(12) $$x_{t}^{\left[k\right]}∼p\left(x_{t}|x_{t-1}^{\left[k\right]},u_{t}\right)$$

The state transition model can be implemented by vehicle motion models, such as kinematic and dynamic vehicle models, that have uncertainty characteristics for the control input and model prediction accuracy.

### 5.2. Updating the Estimate of New Map Features

The next step is to update the estimate of *n*-th new map features $m_{new,n}$ for each particle. The *n*-th new map feature for each particle can be modeled by a Gaussian probability function, as described in (13).

(13) $$m_{new,n}^{\left[K\right]}∼N\left(\mu_{n,t}^{\left[k\right]},\Sigma_{n,t}^{\left[k\right]}\right)$$

Before the update, a map management process is necessary (1) to detect the new physical features that are not registered on the HD map; (2) to infer the existence of the new map feature; and (3) to associate the perceptual measurement with the new map feature.

For the non-associated map features to the measurement $z_{t}$, the estimate of the new map feature is not updated as:

(14) $$<\mu_{n,t}^{\left[k\right]},\Sigma_{n,t}^{\left[k\right]}>=<\mu_{n,t-1}^{\left[k\right]},\Sigma_{n,t-1}^{\left[k\right]}>$$

For the associate map feature to the measurement $z_{t}$, the posterior of the new map feature can be updated using

(15) $$\begin{array}{c} p(m_{new}|x_{1:t},u_{1:t},z_{1:t})\\ =\eta p\left(z_{t}|x_{t},m_{new}\right)p\left(m_{new}|x_{1:t-1},u_{1:t-1},z_{1:t-1}\right) \end{array}$$

where η is the normalization factor. The $p(m_{new}|x_{1:t-1},u_{1:t-1},z_{1:t-1})$ can be represented by Gaussian model of the previous step mean and covariance $N(\mu_{n,t-1}^{\left[k\right]},\Sigma_{n,t-1}^{\left[k\right]})$. The posterior of new map feature $m_{new}$ can be updated based on the linearlization technique of the EKF measurement update for the measurement model $p(z_{t}|x_{t},m_{new})$, as described in

(16) $$\begin{array}{cc} K_{t}^{\left[k\right]} & =\Sigma_{c_{t},t-1}^{\left[k\right]}H_{t}^{\left[k\right]}{\left({H_{t}^{\left[k\right]}}^{T}\Sigma_{c_{t},t-1}^{\left[k\right]}H_{t}^{\left[k\right]}+R_{t}\right)}^{-1}\\ \mu_{c_{t},t}^{\left[k\right]} & =\mu_{c_{t},t-1}^{\left[k\right]}+K_{t}^{\left[k\right]}{\left(z_{t}-{\hat{z}}_{t}^{\left[k\right]}\right)}^{-1}\\ \Sigma_{c_{t},t}^{\left[k\right]} & =\left(I-K_{t}^{\left[k\right]}H_{t}^{\left[k\right]}\right)\Sigma_{c_{t},t-1}^{\left[k\right]}\\ {\hat{z}}_{t}^{\left[k\right]} & =h(x_{t}^{\left[k\right]},\mu_{c_{t},t}^{\left[k\right]})\\ H_{t}^{\left[k\right]} & =\partial h\left(x_{t}^{\left[k\right]},\mu_{c_{t},t}^{\left[k\right]}\right)/\partial x_{t} \end{array}$$

The $c_{t}$ is the index of the associated map feature with measurement $z_{t}$, the *R* is the measurement covariance matrix, the *h* represents the function of the measurement model, the *H* is the Jacobian of the measurement model *h*, and the *K* is the Kalman gain for updating the new map feature.

### 5.3. Importance Weight

The weight $\omega_{t}^{\left[k\right]}$ of each particle should be updated by evaluating the likelihood of perceptual measurement $z_{t}$. For the importance weight of the SLAMCU, there are two types of the measurement likelihoods that one is conditioned on the HD map feature $m_{HD}$ and the other is conditioned on the new map feature $m_{new}$. The weight for the likelihood conditioned on the HD map feature $\omega_{HD,t}^{\left[k\right]}$ can be calculated as

(17) $$\begin{array}{cc} \omega_{HD,t}^{\left[k\right]} & =p(z_{t}|x_{t}^{\left[k\right]},m_{HD})\\ & \approx \eta |2\pi R_{t}{|}^{-\frac{1}{2}}\exp\left\{{\left(z_{t}-{\hat{z}}_{HD,t}^{\left[k\right]}\right)}^{T}R_{t}^{-1}\left(z_{t}-{\hat{z}}_{HD,t}^{\left[k\right]}\right)\right\} \end{array}$$

where ${\hat{z}}_{HD,t}^{\left[k\right]}=h\left(x_{t}^{k},m_{HD}\right)$. The weight for likelihood conditioned on the new map feature $\omega_{new,t}^{\left[k\right]}$ can be calculated as

(18) $$\begin{array}{cc} \omega_{new,t}^{\left[k\right]} & =\eta \int p\left(z_{t}|x_{t}^{\left[k\right]},m_{new}\right)p\left(m_{new}\right)dm_{new}\\ & \approx \eta |2\pi Q_{t}^{\left[k\right]}{|}^{-\frac{1}{2}}\exp\left\{{\left(z_{t}-{\hat{z}}_{t}^{\left[k\right]}\right)}^{T}{Q_{t}^{\left[k\right]}}^{-1}\left(z_{t}-{\hat{z}}_{t}^{\left[k\right]}\right)\right\} \end{array}$$

where $Q_{t}^{\left[k\right]}={H_{t}^{\left[k\right]}}^{T}\Sigma_{c_{t},t-1}^{\left[k\right]}H_{t}^{\left[k\right]}+R_{t}$. The weight $\omega_{t}^{\left[k\right]}$ of each particle can be updated using the equation:

(19) $$\omega_{t}^{\left[k\right]}=\omega_{HD,t}^{\left[k\right]}\times \omega_{new,t}^{\left[k\right]}\times \omega_{t-1}^{\left[k\right]}$$

### 5.4. Resampling

Resampling is performed to randomly generate the new set of particles according to the importance weight of particles. The purpose of the particle resampling is to prevent the weight concentration of the few particles. The resampling is performed only if the following condition is satisfied:

(20) $${\hat{M}}_{eff}=1/\Sigma_{k=1}^{M}\omega_{t}^{\left[k\right]}<\alpha M$$

$M_{eff}$ is the effective number of samples that represents the degree of depletion. When all the particles have even weight values, the $M_{eff}$ has the same value with a number of particles *M*. In contrast, when all the weights are concentrated to a single particle, the $M_{eff}$ has its minimum value of one. The scale factor α is selected according to the probabilistic characteristics of the particle filter.

## 6. Experiments

**Figure 7:**
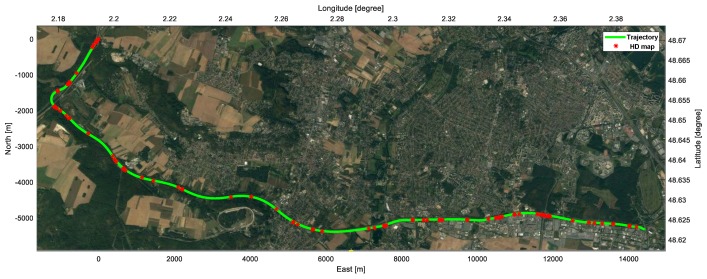
Test sites for the evaluation of the SLAMCU. The green line represents the 20 km-long highway trajectory, the red points shows the position of traffic signs on the HD map.

An experiment was conducted to evaluate the SLAMCU in the real driving situation of 20 km-long highway of France, as shown in Figure 7. The SLAMCU algorithm is able to be applied to many types of features in the HD map, such as lane marking, guardrail, traffic light, and traffic sign. We selected the traffic sign as the validation feature of the SLAMCU because it is straightforward to qualitatively evaluate the proposed algorithm.

### 6.1. Experimental Environment

In order to evaluate the algorithm in the real in-vehicle environment, the traffic signs are measured by a commercial camera which is installed in mass-product vehicles. The camera performs the image processing at 15 FPS to measure the objects in the horizontal view of 40° and the detection range of 80 m. The measurements are transmitted by the CAN bus with 500 Kbps. A HD map produced by HERE offered the position information of the traffic sign features. The traffic signs in the map include lots of information such as position, size, shape, facing orientation, and type. Using the measurements and prior map information, the algorithm performs the RBPF including localization and map change update. The computer with a processor Intel(R) Core(TM) i5-4670 3.40 GHz and 16 GB of RAM takes 30.39 ms on average to process the algorithm.

### 6.2. Existence Inference Based on the DS Theory

**Figure 8:**
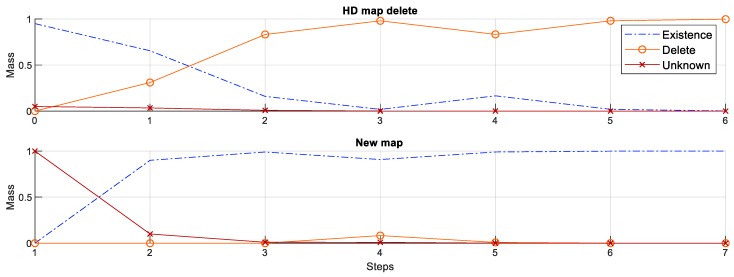
Mass functions of existence state for the deletion of HD map feature and the creation of new map feature.

The changes of the map features are classified based on its existence. The existence could be evaluated by the mass functions of the power set based on the DS theory. Figure 8 shows the mass functions of each existence state according to the sequence of the measurement input for cases of the deletion of HD map feature (upper figure) and the creation of new map feature (lower figure). To determine the deletion of HD map feature, the existence mass functions of the HD map feature was initialized with the (3) and updated based on the Dempster combination rule (5) with the non-measurement existence (2). The existence confidence λ of the traffic sign detection was 0.9 and the existence confidence of HD map feature $\lambda_{HD}$ was 0.95. For the creation of new map feature, the existence mass functions of the new map feature were initialized from the position of the measurement with the (4) and updated based on the combination rule (5) with the measurement existence (1). Although there were outliers for step 4 of the both process, it did not affect the final classification of the map changes.

### 6.3. Classification of the Map Changes

Table 1 shows the confusion matrix to evaluate the classification performance of the SLAMCU for the changes to the HD map. The entire accuracy was 96.12% for the classification of normal, deleted, and new map features. The accuracy of the normal classification was about 98%, and the one missing was due to the visibility occlusion of the traffic sign by a large truck. If the perception sensor is not able to detect the physical features due to the occlusion, there is no way to update the HD map. Therefore, the SLAMCU algorithm automatically does not take into account the occluded features for the update. The classification accuracy of the new map features was 92%, and the wrong classifications occurred because there are not enough measurement sequence of the traffic signs due to the fast vehicle speeds and slow detection of cameras. The map features classified into new and delete were possible to report to the map provider for the reflection of the map changes. For the new map features, SLAMCU estimated the position of the new traffic signs based on the RBPF approach.

### 6.4. Estimation Accuracy of the New Map Features

**Figure 9:**
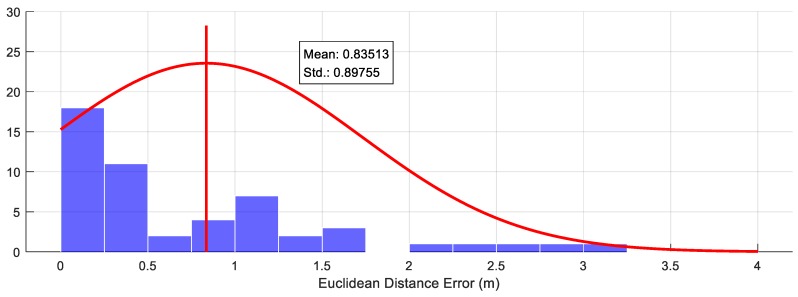
Mapping error for the position estimation of new map features.

The evaluation of position error for the traffic signs classified into the new map feature is shown in Figure 8. The reference position of the new map features for the evaluation obtained by the GraphSLAM based post-processing with real-time Kinematics RTK-GPS. An average of the position error was about 0.8 m and the standard deviation was about 0.9 m, as shown in Figure 9. The position error was caused by the uncertainty of the traffic sign detector using the camera vision. Although there was about one-meter error for the new feature estimation, it is enough to report the location of the map feature change to the map provider and use it as a temporary map feature until the precise ground mapping is performed.

## 7. Conclusions

This paper proposes a simultaneous localization and map change update (SLAMCU) algorithm to detect and update the HD map changes. The SLAMCU is performed using the onboard sensors of individual autonomous cars.

(1) The SLAMCU algorithm applies the evidence (Dempster–Shafer) theory to detect the HD map changes based on the reasoning of the HD map existence. The existence of the map features on the HD map and the new map can be evaluated by Dempster combination rule. Based on the existence inference, the map features can be classified into three classes including the normal, delete, and new map features.
(2) A Rao–Blackwellized particle filter (RBPF) is used to concurrently perform the localization and the SLAM in the SLAMCU framework. The normal HD map features are used to update the localization by matching with the feature perception. The delete HD map is excluded for the localization update in order to prevent the performance degradation. The new map features are updated its position and used to localization based on the RBPF framework. The detected and updated map features are uploaded to the map database of the HD map provider in order to update the map changes of the HD map and share the changes with the other vehicles.
(3) Experiments were performed to evaluate the SLAMCU using on the traffic sign HD map provided by HERE. The experiment results show that the SLAMCU based on the individual cars is sufficient to extract and manage the HD map changes without the special mapping equipment.

This paper presents the SLAMCU process operating in the individual vehicles with validation in the small area. The authors plan to research the integration process of the reported map changes from the multiple SLAMCUs for the wider area of HD map.

## Figures and Tables

**Table 1:** Confusion matrix for classification of the map feature changes.

	Predicted Class					
True class		Normal	Deleted	New	Unclassified	**Accuracy**
	Normal	54	1	0	0	**98%**
	Deleted	0	9	0	0	**100%**
	New	0	0	36	3	**92%**
